# Supplementary material for: Identification of photoperiod-induced specific miRNAs in the adrenal glands of Sunite sheep (Ovis aries)
Source: Front Vet Sci. 2022 Jul 22;9:888207. doi: 10.3389/fvets.2022.888207 (PMC9354845; doi:10.3389/fvets.2022.888207)
Supplement: Supplementary file 1 [file Data_Sheet_1.ZIP › Supplementary file/miRDeep2 file.zip!/miRDeep2 file/Know_Structure/SPLP42A1/oar-mir-376c.pdf]

miRBase precursor : oar-mir-376c
Total read count : 525
oar-miR-376c-5p read count : 226
oar-miR-376c-3p read count : 298
remaining reads : 1

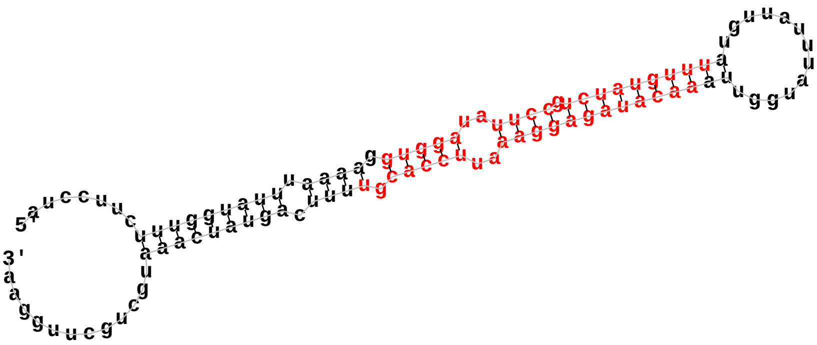

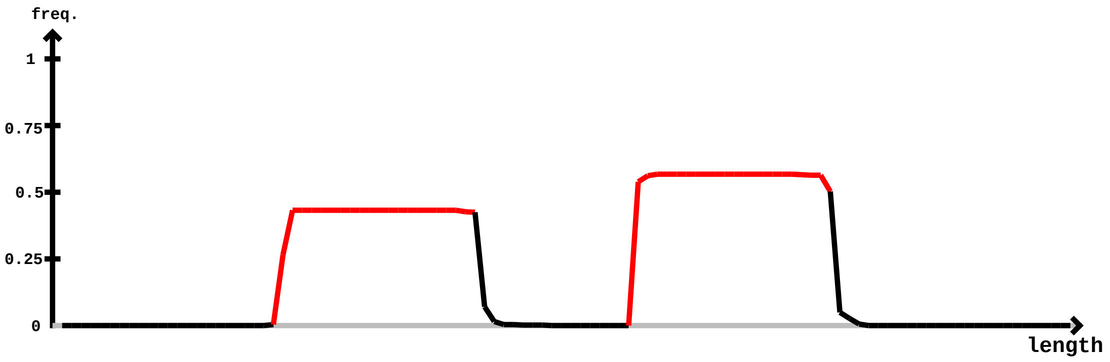


```
                                                                 oar-miR-376c-3p
                        oar-miR-376c-5p
5'- auccuucuuugguauuuaaaagguggauauuccgucuauguuuauguuauuuaugguuaaacauagaggaaauuccacguuuucaguaucaaaugcugcuuggaa  -3'   exp
    .......(((((((((.((((.(((((..((((.((((((((((.............))))))))))))))..))))).)))).))))))))).............  reads   mm   sample
    .....................gguggauauuccgucuauguuu..............................................................  2       0    seq
    ......................guggauauuccgucuaugu................................................................  3       0    seq
    ......................guggauauuccgucuauguu...............................................................  1       0    seq
    ......................guggauauuccgucuauguuu..............................................................  107     0    seq
    ......................guggauauuccgucuauguuua.............................................................  24      0    seq
    ......................guggauauuccgucuauguuuau............................................................  2       0    seq
    .......................uggauauuccgucuauguuu..............................................................  77      0    seq
    .......................uggauauuccgucuauguuua.............................................................  5       0    seq
    .......................uggauauuccgucuauguuuau............................................................  4       0    seq
    .......................uggauauuccgucuauguuuaugu..........................................................  1       0    seq
    .......................uggauauuccgucuauguuuauguuau.......................................................  1       0    seq
    ...........................................................aacauagaggaaauucc.............................  1       0    seq
    ...........................................................aacauagaggaaauucca............................  1       0    seq
    ...........................................................aacauagaggaaauuccacg..........................  32      0    seq
    ...........................................................aacauagaggaaauuccacgu.........................  238     0    seq
    ...........................................................aacauagaggaaauuccacguu........................  11      0    seq
    ............................................................acauagaggaaauuccacguu........................  1       0    seq
    ............................................................acauagaggaaauuccacguuu.......................  8       0    seq
    ............................................................acauagaggaaauuccacguuuu......................  3       0    seq
    .............................................................cauagaggaaauuccacguuu.......................  3       0    seq
```
